# Supplementary material for: Inflammatory factors and risk of lung adenocarcinoma: a Mendelian randomization study mediated by blood metabolites
Source: Front Endocrinol (Lausanne). 2024 Aug 27;15:1446863. doi: 10.3389/fendo.2024.1446863 (PMC11384989; doi:10.3389/fendo.2024.1446863)
Supplement: Supplementary file 1 [file DataSheet1.zip › supplementary files/S2/GCST90200637/forest.pdf]

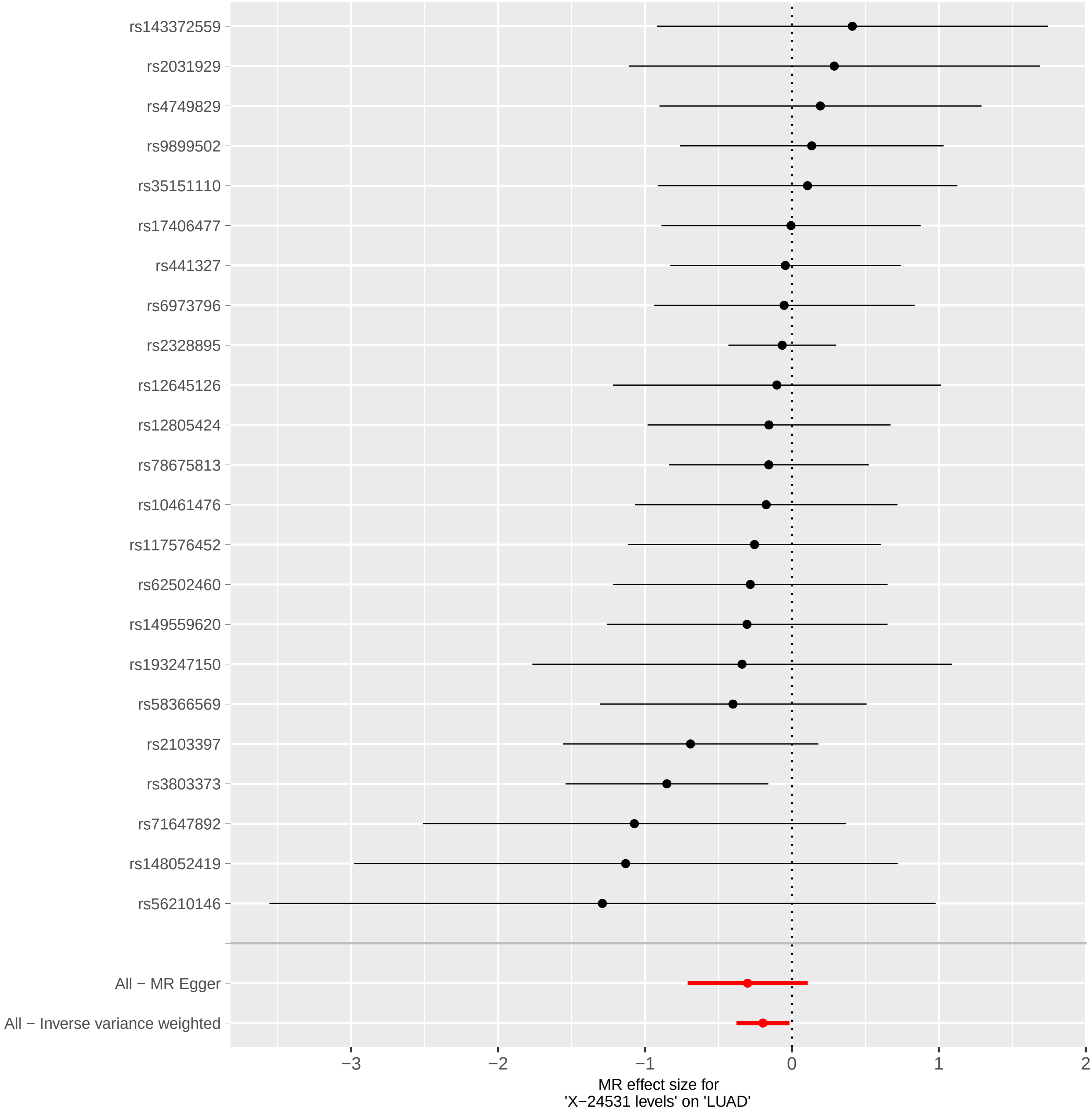
rs143372559
rs2031929
rs4749829
rs9899502
rs35151110
rs17406477
rs441327
rs6973796
rs2328895
rs12645126
rs12805424
rs78675813
rs10461476
rs117576452
rs62502460
rs149559620
rs193247150
rs58366569
rs2103397
rs3803373
rs71647892
rs148052419
rs56210146
All − MR Egger
All − Inverse variance weighted
−3
−2
−1
0
1
2
MR effect size for
'X−24531 levels' on 'LUAD'
